# ICA-Derived EEG Correlates to Mental Fatigue, Effort, and Workload in a Realistically Simulated Air Traffic Control Task

**DOI:** 10.3389/fnins.2017.00297

**Published:** 2017-05-30

**Authors:** Deepika Dasari, Guofa Shou, Lei Ding

**Affiliations:** ^1^School of Electrical and Computer Engineering, University of OklahomaNorman, OK, United States; ^2^Stephenson School of Biomedical Engineering, University of OklahomaNorman, OK, United States

**Keywords:** cognitive factors, time-on-task effect, mental effort, mental workload, EEG, ICA

## Abstract

Electroencephalograph (EEG) has been increasingly studied to identify distinct mental factors when persons perform cognitively demanding tasks. However, most of these studies examined EEG correlates at channel domain, which suffers the limitation that EEG signals are the mixture of multiple underlying neuronal sources due to the volume conduction effect. Moreover, few studies have been conducted in real-world tasks. To precisely probe EEG correlates with specific neural substrates to mental factors in real-world tasks, the present study examined EEG correlates to three mental factors, i.e., mental fatigue [also known as time-on-task (TOT) effect], workload and effort, in EEG component signals, which were obtained using an independent component analysis (ICA) on high-density EEG data. EEG data were recorded when subjects performed a realistically simulated air traffic control (ATC) task for 2 h. Five EEG independent component (IC) signals that were associated with specific neural substrates (i.e., the frontal, central medial, motor, parietal, occipital areas) were identified. Their spectral powers at their corresponding dominant bands, i.e., the theta power of the frontal IC and the alpha power of the other four ICs, were detected to be correlated to mental workload and effort levels, measured by behavioral metrics. Meanwhile, a linear regression analysis indicated that spectral powers at five ICs significantly increased with TOT. These findings indicated that different levels of mental factors can be sensitively reflected in EEG signals associated with various brain functions, including visual perception, cognitive processing, and motor outputs, in real-world tasks. These results can potentially aid in the development of efficient operational interfaces to ensure productivity and safety in ATC and beyond.

## Introduction

Vigilance is indispensable in working environment where automated systems are often used, such as air traffic control (ATC; Warm et al., 2008). Tasks of demanding cognitive workload (Van daalen et al., 2009), along with long working period, can lead to degradation in vigilance, and potentially errors and/or task failure (Danaher, 1980; Dinges, 1995; Warm et al., 2008). In this regard, many studies have been conducted to identify markers that can be used to monitor the evolution of vigilance state and associated behaviors (Ballard, 1996; Smit et al., 2004; Berka et al., 2007; Kim et al., 2017). Such markers can be integrated into human machine interfaces (HMI) to optimize information flow and develop warning systems for situations, in which operators become less vigilant (Parasuraman, 2003; Kramer and Parasuraman, 2007; Parasuraman and Jiang, 2012; Parasuraman et al., 2012).

Task demands, environmental conditions, and engagements are all multidimensional processes that affect operator's state of vigilance. Among them, three mental factors (Davies and Parasuraman, 1982), i.e., mental fatigue, mental workload, and mental effort, have been widely studied. Specifically, mental fatigue, also known as the time-on-task (TOT) effect (Borghini et al., 2014), reflects the unwillingness to perform cognitively demanding tasks (Montgomery et al., 1995). Mental workload is defined as objective task demand imposed on human operators (O'donnell and Eggemeier, 1994; Miyake, 2001), and usually measured as number of successive and/or simultaneous jobs that need to be performed (Wickens, 2015, 2002). Mental effort is a measure of mental capacity that is actually allocated to meet task demands (Paas et al., 2003), and can be measured by number of actions made to accomplish the task (Stone et al., 2005; Rieh et al., 2012). It is also noted that these three factors are interplayed and cannot be completely separated. For example, mental fatigue and effort have been reported to have significant influence on workload (Hockey, 2003). Mental workload is a derivative of task load and environmental factors (i.e., coworkers, noise, etc.), but is also mediated by operators' response to task load that is reflected in operators' mental effort (Endsley and Rodgers, 1998). Increase of mental effort to either meet high mental workload requirement or to mediate mental fatigue-related performance impairment, has been widely reported. Besides, workload variations have been observed to be associated with invested effort and employed strategies (Straussberger, 1997; Veltman and Jansen, 2003).

To measure these three factors in a task, behavioral data are commonly used, e.g., error rate or response time. However, such data can only be recorded at discrete time instants, which is not suitable for continuous monitoring of the evolution of these mental factors. On the contrary, electroencephalography (EEG) that can continuously measure brain activities has gained increasing attention in this field (Berka et al., 2004; Fink et al., 2005; Craig et al., 2012), and has been indicated as a reliable technique for characterizing mental state changes of operators at the resolution of second or sub-second level (Ray and Cole, 1985; Klimesch, 1996; Smith and Gevins, 2005). In these studies, EEG correlates of spectral powers at channel domain have been examined with various mental factors, such as mental workload (Sterman et al., 1993; Sterman and Mann, 1995; Berka et al., 2005; Helton and Russell, 2011; Galy et al., 2012; Aricò et al., 2016b), mental effort (Ullsperger et al., 1988; Miyata et al., 1990; Backs and Seljos, 1994; Smit et al., 2004), and mental fatigue (Boksem et al., 2005; Smith and Gevins, 2005; Craig et al., 2012; Borghini et al., 2014). Identified EEG correlates are frequency and region dependent (Sterman et al., 1993; Brookings et al., 1996; Makeig and Jung, 1996; Gevins and Smith, 1999; Wilson, 2002). For example, theta band power changes were usually found at the frontal midline channels to be linked to the development of mental fatigue (Yamamoto and Matsuoka, 1990; Gevins et al., 1995; Onton et al., 2005; Chai et al., 2016), variations of mental effort (Miyata et al., 1990), and mental workload (Yamamoto and Matsuoka, 1990; Gundel and Wilson, 1992). Alpha band power changes sensitive to complex motor function (Pfurtscheller et al., 1994), mental workload, and mental effort in attentive stimulus processing and expectancy (Ray and Cole, 1985; Keil et al., 2001; Lin et al., 2010) were identified over the centro-parietal and parietal areas (Gevins et al., 1995; Gevins and Smith, 1999; Borghini et al., 2016; de Vries et al., 2017). Alpha power changes are also associated with reduction in attention with TOT (Klimesch, 1999; Schier, 2000). Some researchers also investigated various indices based on beta band power and/or ratio of beta band power to either alpha or theta band power (Eoh et al., 2005). Among these studies, the majority used a classical cognitive paradigm with repetitive stimuli (Ullsperger et al., 1988; Keil et al., 2001; Boksem et al., 2005; Onton et al., 2005; Lin et al., 2010). Although such tasks can facilitate subsequent data analysis, e.g., event related potential (ERP) analysis (Trejo et al., 1995; Lorist et al., 2000; Smith and Gevins, 2005; Galy et al., 2012), they are limited in the investigation of intrinsic dynamics of mental factors in real-world tasks. In real-world tasks, operators work in environments with a variety of visual and auditory stimuli that continuously and dynamically change with the inputs and/or responses. Some studies have tried to simulate real-world task situations (e.g., driving, aircraft landing, and takeoff, etc.) (Sterman et al., 1993; Gevins et al., 1995; Sterman and Mann, 1995; Brookings et al., 1996; Wilson, 2002; Kohlmorgen et al., 2007; Craig et al., 2012; Aricò et al., 2016a; Blankertz et al., 2016) with varied levels of task difficulty and/or load to examine EEG correlates accordingly. However, these tasks are still a little far from real-world tasks. Furthermore, most of previous studies examined EEG correlates of mental states and/or behaviors on EEG signals at channels (Sterman et al., 1993; Boksem et al., 2005; Borghini et al., 2014). However, EEG signals at channels are believed to be mixed from multiple neural sources due to the volume conduction effect (Wolters and de Munck, 2007) and, therefore, less indicative to underlying neural substrates. Among various methods (Jung et al., 2000; Tenke and Kayser, 2005; Cavanagh et al., 2009; Ding, 2009; Liao et al., 2012; Zhu et al., 2014) to dissociate EEG signals into component signals that are more indicative to underlying neural substrates, independent component analysis (ICA) (Onton et al., 2005) has demonstrated great success (Onton et al., 2006; Shou et al., 2012), which is promising to analyze continuous EEG data from real-world tasks.

The present study was conducted to directly address the challenge of complexity in real-world tasks through technique advancement. We proposed to use the ICA method to precisely probe EEG correlates to three major mental factors, i.e., mental fatigue, mental workload, and mental effort, in a real-world task i.e., a realistically simulated ATC task (Bailey et al., 1999), which was used to train ATC officers in Federal Aviation Association (FAA). The ATC task required operators to continuously search, select, and integrate information from multiple sources to make decisions for efficient air traffic flow. Furthermore, the ICA technique was used to decompose recorded EEG data into different component signals, indicative of specific neural substrates, which was novel since it enabled the investigation of impact of three mental factors on identified neural substrate functions. We specifically examined EEG correlates of selected ICs' spectral power changes at their dominant frequency bands to different levels of mental factors.

## Materials and methods

### Participants

Ten subjects (age 25 ± 4.3, all males and right handed) participated in this study after giving written informed consent. Among them, two subjects were excluded since their performance was significantly different from others (Table 1). Experimental protocol was approved by the institutional review board (IRB) at the University of Oklahoma.

**Table 1:** **Summary of perfromacne and task related metrics from 20 sessions of 10 subjects**.

**Subject and session (S#s#)**	**Number of warnings**	**Number of crashes**	**Number of clicks per minute**	**Average number of aircraft per minute**	**Correlation (R, *p*)**
S1s1	0	2	21.14 ± 6.63	3.25 ± 0.57	0.18, 0.06
S1s2	0	0	24.56 ± 7.57	3.41 ± 0.63	0.56, <0.001
S2s1	6	4	22.05 ± 4.16	3.28 ± 0.64	0.37, <0.001
S2s2	9	3	23.55 ± 5.63	3.50 ±0.66	0.46, <0.001
S3s1	12	0	20.77 ± 6.62	3.71 ± 0.69	0.18, 0.06
S3s2	18	2	23.49 ± 8.32	3.80 ± 0.71	0.25, <0.01
S4s1	10	2	18.97 ± 5.13	3.63 ± 0.61	0.35, <0.001
S4s2	2	1	19.25 ± 4.67	3.52 ± 0.67	0.4, <0.001
S5s1	13	4	19.95 ± 5.64	4.41 ± 0.88	0.39, <0.001
S5s2	4	0	18.23 ± 4.98	3.61 ± 0.60	0.46, <0.001
S6s1	23	9	23.14 ± 5.85	4.01 ± 0.81	0.23, <0.05
S6s2	10	4	23.77 ± 6.13	3.67 ± 0.65	0.37, <0.001
S7s1	9	5	24.56 ± 5.17	4.06 ± 0.78	0.20, <0.05
S7s2	9	1	24.87 ± 5.45	3.74 ± 0.65	0.51, <0.001
*S8s1*	416	94	*16.24 ± 4.74*	7.56 ± 3.12	0.09, 0.34
*S8s2*	320	83	*17.33 ± 5.39*	8.38 ± 2.74	0.13, 0.18
*S9s1*	180	73	*15.87 ± 5.39*	6.12 ± 2.07	−0.04, 0.60
*S9s2*	94	41	*17.18 ± 5.31*	4.25 ± 1.27	0.08, 0.38
S10s1	23	11	24.92 ± 5.53	4.17 ± 1.10	0.38, <0.001
S10s2	2	0	20.10 ± 5.95	3.52 ± 0.63	0.51, <0.001

*Here S# represents the subject number and s# represents the session number. The last column indicates correlation coefficient values and associated p-values between the number of active aircraft and the number of clicks. The subjects with bad performance are marked in italic*.

### Experimental procedures

All the experiments were conducted in an electromagnetic shielding room. Subjects were continuously monitored for their engagement in the task through a one-way mirror by the experimenter.

**Figure 1:**
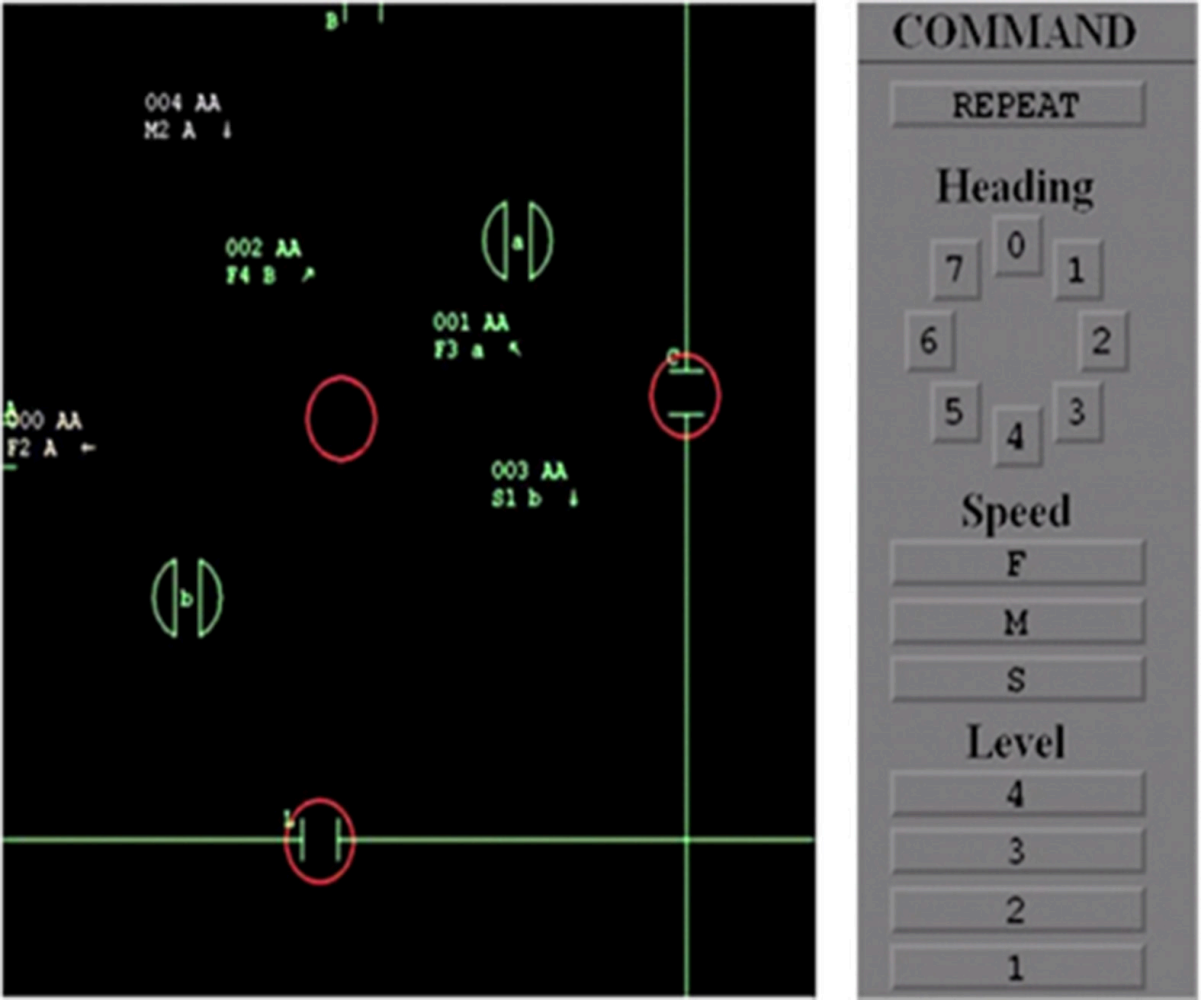
**The air traffic control task interface for C-Team**. Control panel (the gray box on the right) with controls for heading direction, speed, and level of aircraft. The aircraft (represented by arrows), airports (represented by lowercase letters with two half circles), exits (represented by uppercase letters), and restricted areas (represented by red circles). White and green colors denote aircrafts that are in active and en route respectively.

The ATC scenarios were simulated with CTEAM V2.0 software (Bailey et al., 1999), which provided a realistically simulated ATC task for training ATC officers without any modification for the present study, and shown on a 21-inch LCD monitor at a distance of 50 inches from the subject. The CTEAM interface consisted of an airspace area and control panel (see Figure 1). The control panel had buttons to control heading, speed, and altitude (level). The airspace area contained two airports (represented as a, b), two exits (represented as A, B) and three restricted areas marked as red circles. Subjects were required to activate (represented in green color) and then navigate aircraft to their destination (i.e., either an airport or an exit) as specified by the data block on the aircraft using control actions. Subjects were required to perform control actions, i.e., changing heading, speed, and altitude of an aircraft, to regulate and maintain efficient air traffic flow in virtual airspace using mouse. The ATC task lasted 2 h with air traffic flow at a rate of two aircrafts per minute. Numbers of control actions performed and errors (i.e., proximity warnings and crashes), and scenario status (e.g., number of active and inactive aircraft in airspace) were automatically logged into a replay file every 5 s.

All subjects were trained in a training session, which included watching a 5 min demo, and performing a 30 min scenario to familiarize themselves with the task and develop strategies for completing the task. After the training, all subjects performed two 2 h recording sessions on different days during which EEG data were collected using a Net Amps 300 Amplifier (Electrical Geodesics, Inc., Eugene, OR) with 128 channels, which provided a high spatial resolution. The data were collected at a sampling rate of 250 Hz. Electrode impedances were ensured to be below 50 kΩ before each session. To ensure low impedance throughout the session, EEG recording was paused at 1 h time mark and, at the electrodes with impedance higher than 50 kΩ, electrolyte solutions were used to reduce impedance. During this process, subjects were instructed to continuously perform the task.

### Data analysis

#### Behavioral and performance data analysis

Each subject's performance was evaluated by extracting number of errors (i.e., number of crashes and warnings) from the replay files. In addition, average number of active aircraft in airspace per minute and number of inputs (i.e., number of mouse clicks) per minute were also extracted from logged data, as the behavioral measures for mental workload and mental effort, respectively.

#### EEG preprocessing

EEG data were preprocessed using the Net Station Software (Electrical Geodesics, Inc., OR, USA). After applying a band-pass filter of 0.5–30 Hz, noisy channels were identified if a channel has more than 20% of data above an amplitude threshold of 200 μV over the entire recording and then replaced with data interpolated from neighboring channels using a spherical spline method from the Net Station Software (Perrin et al., 1989). EEG data were then down-sampled to 125 Hz to reduce computational loads. Extended Infomax ICA (Lee et al., 1999) from the EEGLAB toolbox was performed for artifacts rejection. Artifactual ICs were identified using: (1) the ADJUST software (Mognon et al., 2011) to automatically identify ICs related to eye blink, vertical/horizontal eye movement, and generic discontinuity; (2) visual inspection to identify ICs related to electromyogram (EMG) and electrocardiogram (ECG). All artifactual ICs identified were removed to obtain artifact-free EEG data for subsequent analyses.

#### Group-level ICA

A group-level ICA method was implemented on artifact-free EEG data that were temporally concatenated across all subjects and sessions (Delorme and Makeig, 2004). To reduce amount of EEG data for the group-level ICA analysis, global field power (GFP) data at each time point were computed as standard deviation of the momentary potential values across all channels (Lehmann and Skrandies, 1980). The GFP data were further transformed into *z*-values and the ones larger than four were removed (Yuan et al., 2012). In the remaining GFP data, both local peaks and troughs were representative of stable functional states spanning from around 50 to 100 milliseconds (ms) (Pascual-Marqui et al., 1995). EEG data at time instants corresponding to these local GFP peaks were selected for each session to choose EEG data with relatively high SNRs (Skrandies, 1990) and were temporally concatenated across all sessions for the subsequent ICA analysis. As the first step of ICA, principle component analysis (PCA) was used to reduce the data dimension to 64, which was determined as an optimal number to disentangle brain related ICs from artifact ICs in a preliminary investigation using different numbers of components (i.e., 25, 32, 64, etc.). Then an extended Infomax ICA algorithm was used to obtain ICs (Lee et al., 1999), which has been demonstrated with better performance than the standard Infomax algorithm (Di Flumeri et al., 2016). From 64 ICs, ICs of interest were selected based on two criteria (Shou and Ding, 2014): (1) spatial pattern: scalp map of the IC that could be approximately explained by one or two dipolar sources (Delorme et al., 2012); (2) spectral pattern: power spectrum density (PSD) of ICs exhibiting peaks in the theta (4–<8 Hz) and/or alpha (8–<12 Hz) bands.

#### Analysis of selected ICs

**Figure 2:**
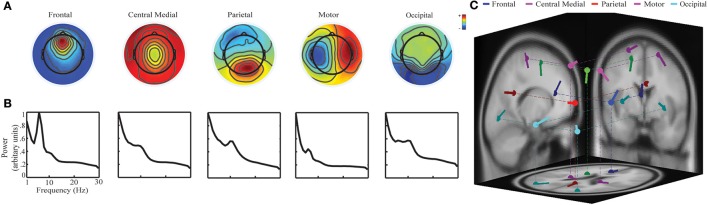
**Five brain activity related ICs identified using group-level ICA: (A)** spatial scalp maps; **(B)** averaged PSDs; and **(C)** dipole source locations on the MNI template.

For the selected ICs, two types of analyses were sequentially performed. Firstly, to investigate plausible neural substrates for selected ICs, dipolar source localization was performed to fit scalp maps of ICs using DIPFIT in EEGLAB (Delorme and Makeig, 2004). Talairach coordinates of the fitted dipole sources were identified and further associated with Brodmann areas (BAs). Secondly, ICs' spectral powers were calculated using short-time Fourier transform (STFT) on each 1-s segment ranging from 1 to 30 Hz. For each IC, only spectral powers at its dominant frequency band, i.e., the evident peak in PSD (see Figure 2), were examined to investigate their relationship to three mental factors.

#### Identifying EEG correlates to TOT effect

Two different methods, i.e., regression and bin analyses, were used to examine the relationship between individual IC's spectral powers and TOT effect. Firstly, linear regression analyses were performed to examine power changes with TOT in both individual session level and group levels (Shou and Ding, 2013), at a resolution of 1 min over 100 min after excluding 20 min period of impedance check (usually took <10 min). In the group level, 1 min data from individual sessions was firstly averaged across all sessions and then imported for linear regression analysis. Then, to test whether the pattern was significantly detected over all sessions and subjects, the number of significant regression models with same slope types (either positive or negative) were examined across all sessions by a binomial test (as compared to total number of sessions, i.e., 16). Secondly, motivated by previous studies on the TOT effect (Trejo et al., 2007; Cheng and Hsu, 2011), a bin analysis was performed at four 5 min time windows, i.e., the beginning period from 5 to 10 min (labeled as T1), the pre-impedance check period from 35 to 40 min (labeled as T2), the post-impedance check period from 75 to 80 min (labeled as T3), and the ending period from 95 to 100 min (labeled as T4). ICs' spectral powers were compared using a repeated measure ANOVA test. Additionally, independent *post-hoc* paired *t*-tests of powers between two paired bins were performed to determine these time periods of significant changes due to the TOT effect.

#### Identifying EEG correlates to mental workload and effort

Bin analyses were performed to identify ICs with their dynamics that were significantly correlated to mental workload and mental effort. A bin analysis on residual spectral power data after subtracting regressed TOT-related power data (Shou and Ding, 2013) was performed, since significant regression models for the TOT effect were identified in most sessions. Specifically, two bins of IC power data were obtained at the highest 30% and the lowest 30% data from the number of clicks (indicating mental effort) or the number of active aircraft (indicating mental workload). Paired *t*-tests were performed to compare data in two bins. For the purpose of comparison, the same analysis was also performed on data without removing TOT-related power changes. The *p*-value was set at 0.05 with Bonferroni correction.

## Results

### Behavioral and performance measures

All subjects (represented as S#, where # indicates subject number) finished two sessions of the task (i.e., s1 and s2), with two have significant outlier performance (i.e., S8 and S9, Table 1). All the remaining subjects had reasonable performance with low number of proximity warnings (9.37 ± 7.26, mean ± SD) and crashes (3.0 ± 3.20). While S8 and S9 showed significantly worse performance (number of crashes: from 41 to 94; number of proximity warnings: from 94 to 416), they were successful in navigating most aircraft and dealing with most warnings. Moreover, the numbers of active aircraft (3.70 ± 0.32) was also consistent in the sessions across all eight subjects. It was also observed that the number of active aircraft and clicks had a significant positive correlation in 14 sessions out of 16 sessions analyzed (last column in Table 1). The number of significant positive correlation was significantly detected across all sessions based on the binomial test (*p* < 0.05). Since the performance data of S8 and S9 were significantly different from others, they were excluded from the following EEG analysis.

### Brain activity related ICs

Five ICs indicating prominent characteristics of brain activity were selected (Figure 2A). They were labeled as the frontal IC, the central medial IC, the parietal IC, the motor IC, and the occipital IC, according to their spatial patterns and locations of fitted dipole sources (Makeig et al., 2002; Onton et al., 2005; Shou et al., 2012). In the spectral patterns (Figure 2B), i.e., PSD, the frontal IC showed an evident peak in the theta band while the other four ICs had evident peaks in the alpha band. Therefore, these bands have been selected as the dominant frequency band for each IC, respectively. In the source location (Figure 2C), the frontal IC had the topography of a radial source over the frontier area with its fitted dipole source in the frontal cortex [Talairach coordinate: (−3, 30, 11); BA 24; residual variance: 8.24%]. The central medial IC had the topography of a radial source with its fitted dipole over the supplementary motor area (SMA) [(−6, −10, 43); BA 6; 5.15%]. The parietal IC had the topography of a radial source over the parietal area with its fitted dipole into the dorsal posterior parietal cortex (PCC) [(4, −47, 28); BA 23; 1.24%]. The motor IC showed a tangential dipolar pattern over bilateral primary motor cortices [(−23, −21.61) and (23, −21, 61), BA 4; 4.92%]. The occipital IC had a tangential dipolar pattern over bilateral occipital cortices [(−32, −78, 5) and (32, −78, 5), BA 18, 3.88%].

### IC power changes with TOT

**Figure 3:**
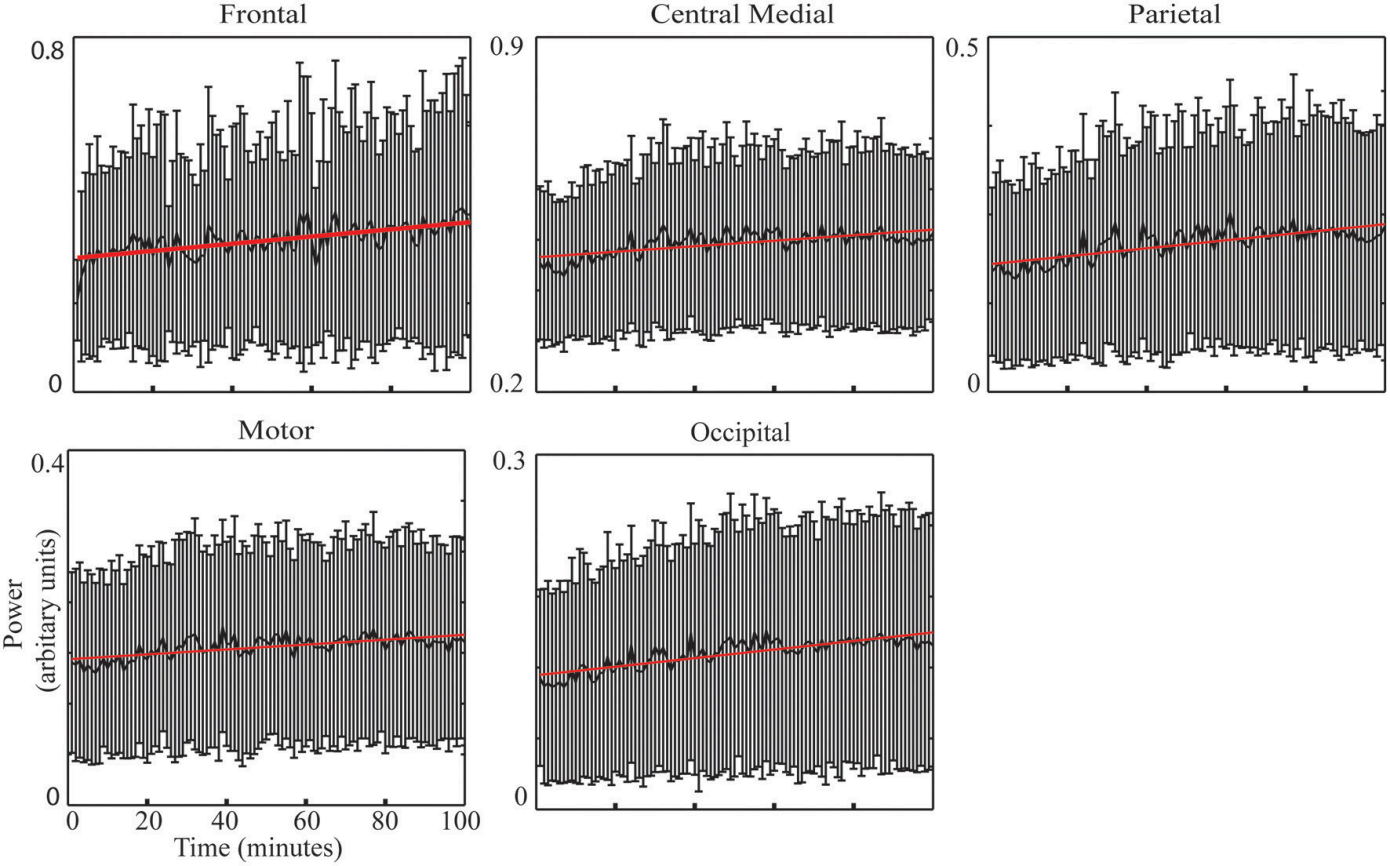
**IC spectral powers across 100 min (thick black curve: mean) across all sessions with the linearly regressed lines (red line)**.

**Figure 4:**
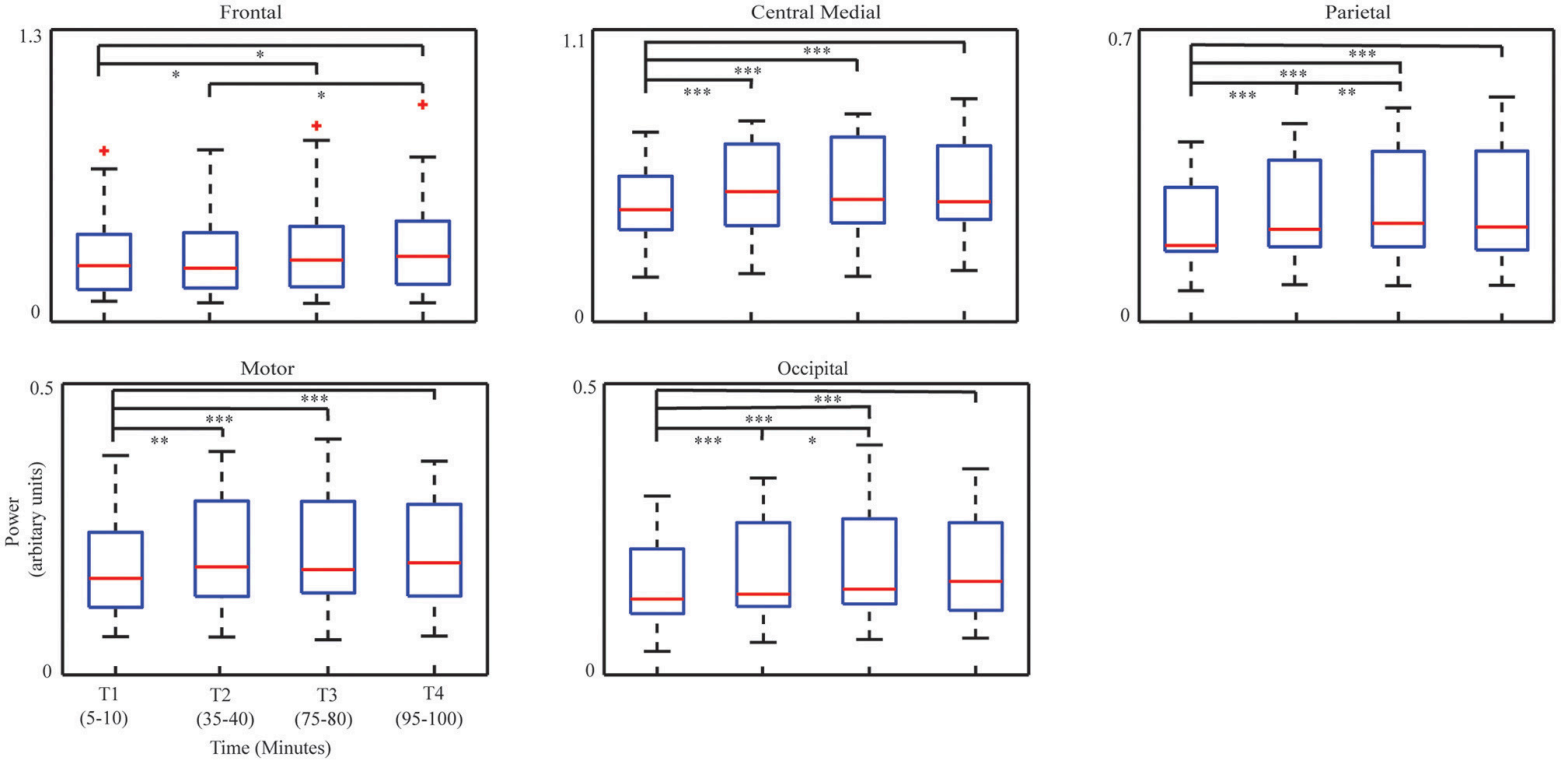
**Whisker plots of averaged IC powers at corresponding dominant freqency bands in four time periods**. Median: red central mark; 25th and 75th percentiles: lower and upper edges of the box, respectively; maximum whisker length: 1.5, and the outliers: red cross. ^*^p < 0.05, ^**^p < 0.01, and ^***^p < 0.001.

Figure 3 illustrates the group-level regression models of spectral powers at five selected ICs. It is noted that all five ICs' spectral powers significantly increased as TOT (*p* < 0.05). In the individual-level regression models, 13 sessions were observed to have significant regression models with positive slopes in the frontal IC (*p* < 0.05, Table 2). Similar pattern of significant regression models with positive slopes were detected in the other four ICs (Table 2), i.e., 12 sessions in the central medial IC, 12 sessions in the motor IC, 14 sessions in the parietal IC, and 13 sessions in the occipital IC. The number of positive slopes was detected of significance (*p* < 0.05) by the binomial tests in all ICs. Figure 4 illustrates IC powers at four different time periods.

**Table 2:** **Summary of linear regression analysis of IC power dynamics at corresponding dominant frequency bands of each session at each subject**.

**Subject and session (S#s#)**		**Frontal Theta**		**Central Medial Alpha**		**Parietal Alpha**		**Motor Alpha**	**Occipital Alpha**	
	**Slope**	***p***	**Slope**	***p***	**Slope**	**P**	**Slope**	***p***	**Slope**	***p***
S1s1	+	<0.001	+	<0.001	+	<0.001	+	<0.001	+	<0.01
S1s2	+	<0.001	+	<0.001	+	<0.001	+	<0.05	+	<0.001
S2s1	+	<0.001	+	<0.001	+	<0.01	+	<0.01	−	0.31
S2s2	+	0.43	+	<0.001	+	<0.01	+	<0.05	+	0.22
S3s1	+	<0.001	+	0.19	+	<0.001	+	<0.05	+	<0.001
S3s2	+	<0.05	+	<0.001	+	<0.001	+	0.23	+	<0.001
S4s1	+	<0.001	+	<0.001	+	<0.001	+	<0.001	+	<0.001
S4s2	+	<0.05	+	0.64	+	<0.01	+	<0.05	+	<0.001
S5s1	+	0.5	+	<0.001	+	<0.001	+	<0.001	+	<0.001
S5s2	+	<0.01	+	<0.05	+	<0.001	+	<0.001	+	<0.01
S6s1	+	<0.001	+	<0.001	+	<0.001	+	<0.001	+	<0.01
S6s2	+	<0.001	+	<0.01	+	<0.001	+	<0.001	+	<0.001
S7s1	−	<0.01	−	<0.001	−	0.6	−	0.96	−	0.47
S7s2	+	<0.001	+	<0.001	+	<0.001	+	<0.001	+	<0.001
S10s1	+	<0.001	+	<0.01	−	0.94	+	0.64	+	<0.001
S10s2	+	<0.001	−	0.31	+	<0.001	+	0.14	+	<0.001

*“+”: positive slope and “−”: negative slope*.

The ANOVA analysis on four bins' data revealed consistent increasing patterns with the regression analysis in all ICs, i.e., the frontal IC (*F* = 5.28, *p* < 0.001), the central medial IC (*F* = 13.15, *p* < 0.001), the parietal IC (*F* = 18.12, *p* < 0.01), the motor IC (*F* = 10.52, *p* < 0.001), and the occipital IC (*F* = 15.03, *p* < 0.001). In the paired *t*-tests, compared to T1, significant increases of powers were detected in T3 (*p* < 0.05, *t* = −2.56) and T4 (*p* < 0.05, *t* = −2.60) in the frontal IC, as well as in the central medial, parietal, motor, and occipital ICs [the central medial IC: T2 (*p* < 0.001, *t* = −4.22), T3 (*p* < 0.001, *t* = −4.09), T4 (*p* < 0.05, *t* = –4.61), the parietal IC: T2 (*p* < 0.001, *t* = −4.71), T3 (*p* < 0.001, *t* = −5.10), T4 (*p* < 0.001, *t* = −4.40); the motor IC: T2 (*p* < 0.01, *t* = −3.42), T3 (*p* < 0.001, *t* = −4.26), T4 (*p* < 0.001, *t* = −4.10); the occipital IC: T2 (*p* < 0.001, *t* = −4.41), T3 (*p* < 0.001, *t* = −4.50), T4 (*p* < 0.001, *t* = −5.10)]. In addition, significant increases of powers were detected between T2 and T3 in the parietal IC (*p* < 0.01, *t* = −3.22) and in the occipital IC (*p* < 0.05, *t* = −2.14) and between T2 and T4 in the frontal IC (*p* < 0.05, *t* = −2.61).

### IC power changes with mental effort

**Figure 5:**
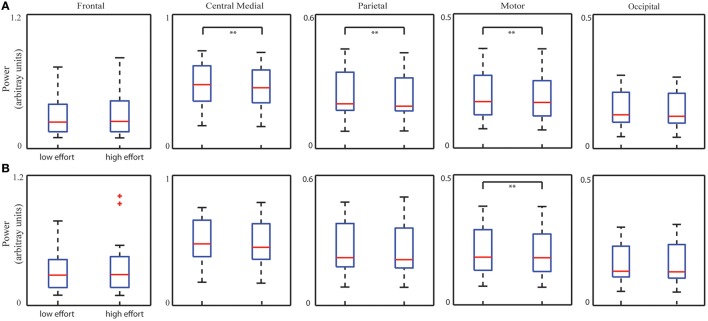
**Whisker plots of averaged IC PSDs at corresponding dominant freqency bands from all the sessions of all subjects in low and high mental effort conditions with (A)** or without **(B)** removing TOT-related PSD changes. ^**^p < 0.01, corrected.

Figure 5 illustrates five ICs' spectral powers for two bins corresponding to low and high levels of mental effort as measured by the number of clicks per minute (low bin: 15.47 ± 2.11 and high bin: 29.08 ± 3.03; *p* < 0.001, *t* = −21.53). Statistical analysis on spectral powers of the ICs after removing the TOT-related effect (Figure 5A) with the Bonferroni correction revealed significant decrease in three ICs (the central medial IC: *p* < 0.01, *t* = 3.65; the parietal IC: *p* < 0.01, *t* = 3.85; the motor IC: *p* < 0.01, *t* = 3.60). A significant increase in the frontal IC spectral power (*p* < 0.05, *t* = −2.29) was also observed when the mental effort increased, however, it did not survive after the Bonferroni correction. As a comparison, the bin analysis results on data without removing TOT effect were also displayed in Figure 5B, i.e., a significant decrease only in the motor IC (*p* < 0.01, *t* = 2.97) in the contrast of high level and low level mental effort after Bonferroni correction.

### IC power changes with mental workload

**Figure 6:**
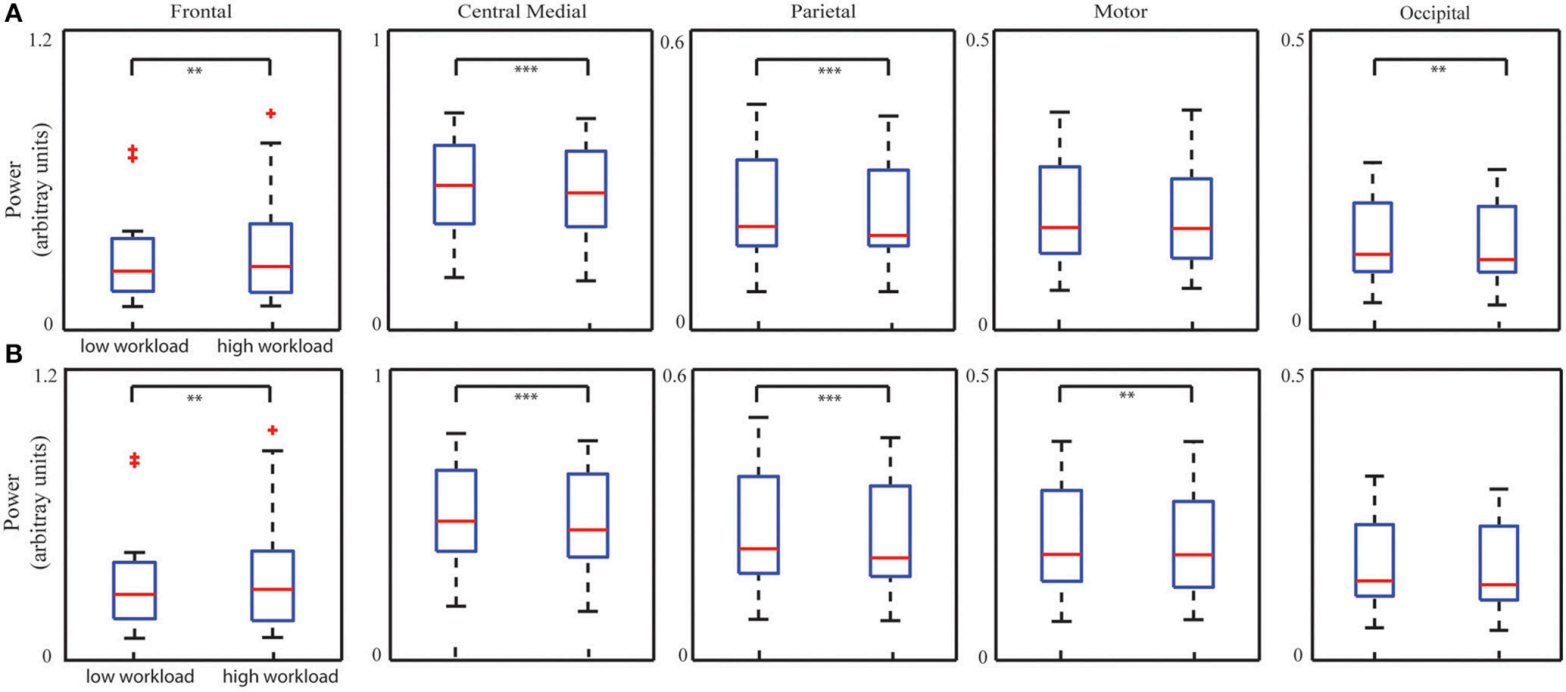
**Whisker plots of averaged IC PSDs at corresponding dominant freqency bands from all the sessions of all subjects in low and high mental workload conditions with (A)** or without **(B)** removing TOT-related PSD changes. ^**^p < 0.01, and ^***^p < 0.001, corrected.

Figure 6A illustrates five IC's spectral power for two bins corresponding to low and high levels of mental workload as measured by the number of active aircraft (low bin: 37.56 ± 2.94 and high bin: 58.01 ± 6.10; *p* < 0.001, *t* = −21.42). Statistical analysis on spectral powers of the ICs after removing TOT effect (Figure 6A) revealed a significant increase from the low level to the high level in the frontal IC (*p* < 0.01, *t* = −3.31) and significant decreases in the other three ICs (the central medial IC: *p* < 0.001, *t* = 4.34; the parietal IC: *p* < 0.001, *t* = 4.95; the occipital IC: *p* < 0.01, *t* = 3.01), after Bonferroni correction. As a comparison, the bin analysis results on data without removing the TOT effect were also displayed in Figure 6B, i.e., significant increase in the frontal IC (*p* < 0.01, *t* = −2.95), and significant decrease in other three ICs (the central medial IC: *p* < 0.001, *t* = 4.89; the parietal IC: *p* < 0.001, *t* = 4.65; the motor IC: *p* < 0.01, *t* = 2.96) in contrast of high level and low level mental workload, after Bonferroni correction.

## Discussion

In the present study, EEG component signals that coded information about three mental factors, i.e., TOT (or mental fatigue), mental workload, and mental effort, were examined in a realistically simulated ATC task. In the task, complex visual and auditory information were continuously streamed to subjects (Dittmann et al., 2000), forming a largely different task protocol in studying mental factors as compared to classical cognitive tasks, in which simple and repeated stimuli are generally used (Boksem et al., 2005; Berka et al., 2007; Helton and Russell, 2011). Such a task protocol also enforces strict requirements on data analysis when probing underlying neural sources and their dynamics over time. A data-driven ICA method was used to disentangle EEG signals into different component signals at group level. Significant relationships between spectral powers of identified component signals at dominant frequency bands and mental factors were successfully elucidated.

### Neuronal processes underlying ICs

EEG spectral power correlates to different mental factors were examined in the IC domain rather than the channel domain, with two purposes: (1) to reduce volume conduction effect (Shou and Ding, 2014); (2) to examine relationship between spectral power dynamics from specific neural substrates related to different mental factors. Among five identified ICs (Figure 2), the frontal IC with the dominant theta band power revealed a probable neural generator in the anterior cingulate cortex (ACC) area, which has been extensively reported in many cognitive tasks that require concentration, attention, performance monitoring, and short-term memory (Ishii et al., 1999; Jensen and Tesche, 2002; Onton et al., 2005; Klimesch et al., 2007; Scheeringa et al., 2009). Such neurocognitive resources are largely demanded in the ATC task, since operators need to continuously respond to appearance of aircraft, design optimal flight routines, monitor aircraft, and avoid potential warnings and/or crashes. The central medial and motor ICs with dominant alpha band activity were localized to motor related areas, i.e., primary and supplementary motor areas, that have been associated with motor planning and execution (Pfurtscheller et al., 1994; Pfurtscheller and Da Silva, 1999), which can be associated with actions performed by operators using input devices (i.e., mouse) during the task (Grinband et al., 2011). The parietal IC with its neural generator within the PCC represents sensory information evaluation and integration for planned movements (Polich, 2007). The occipital IC with its neural generator in bilateral visual cortices represents visual perception and processes in the task (Stewart et al., 2014).

### Neural mechanisms related to TOT

TOT is revealed in the dynamics of all five ICs with similar increasing pattern (Figure 3 and Table 2), suggesting that TOT is a top-down process and generates progressive effects on most brain functions. Consistent with previous findings (Makeig and Jung, 1996; Lorist et al., 2000; Boksem et al., 2005; Craig et al., 2012), enhanced alpha powers indicate insufficient suppression of alpha rhythms during sensory integration (the parietal IC and the occipital IC) and motor outputs (the motor and central medial ICs), which causes reduced effectiveness in these neural processes with the development of mental fatigue (Lorist et al., 2000; Boksem et al., 2005). Enhanced theta power in the frontal IC may indicate a counterbalance mechanism by recruiting more cognitive resources to combat performance degradation induced by TOT (Klimesch et al., 1997).

### Neural mechanisms related to mental workload and mental effort

Mental effort and mental workload derived from the behavioral metrics also depict significant variations in spectral powers of most ICs. Enhanced theta power from the frontal IC was observed in the bin of high-level mental workload (tendency also identified in the bin of high-level mental effort while not significant after correction), whereas in general reduced alpha powers from the other four ICs in the bin of high-level mental effort/workload were observed. Increase in the frontal theta power reflects more cognitive resource allocations to produce response actions, i.e., mouse clicks (or mental effort; Gundel and Wilson, 1992). Reduced alpha powers is observed with statistical significance in other ICs in both bins of high-level mental effort and workload. It is indicative of increased engagements of the motor cortices in motor planning and execution (Sterman et al., 1993; Gevins and Smith, 1999) and parietal and occipital cortices in sensory information integration (Pfurtscheller et al., 1994; Keil et al., 2001; Stewart et al., 2014). The fact that similar patterns are identified between these two mental factors and EEG ICs dynamics is consistent with the observation of significant correlations between these two measures from behavioral/performance data (Table 1). While similarity in general, it is noted that the significant alpha power change in the motor IC is only observed in the analysis of mental effort (Figure 5A) and the significant alpha power change in the visual IC is only observed in the analysis of mental workload (Figure 6A). This might be explained by the fact the behavioral measure for mental effort (i.e., number of clicks) is more related to the motor function while the behavioral measure for mental workload potentially requires more visual scanning due to increased number of active aircraft. Together with this evidence, more detections of significant changes of IC spectral powers in the analysis of mental effort suggest IC power spectral dynamics after removing the TOT effect might reflect realistic dynamics in two used behavioral measures. In the analysis of mental workload, significant changes that are observed in general both before and after removing the TOT effect, might suggest its stronger influences on IC power spectral dynamics, which are less smeared by the TOT effect. The only detection of tendency of significant changes on the frontal IC theta power in the analysis of mental effort also support the notion that the influences of mental effort (measured by number of clicks) are less strong than those of mental workload (measured by number of active aircraft).

### Limitations

Several limitations of the present study must be noted as precautions in the interpretation of our data. Firstly, given the low number subjects, the results reported are only exploratory and can be useful in elaborating the working hypothesis in further studies with real-world ATC scenarios performed by experienced ATC operators. Secondly, an interruption of the task at 1 h to monitor the impedance of channels could have resulted in artifacts such as EMG in the present study. While a pre-processing step to remove artifacts based on ADJUST and visual inspection was implemented, such factors might still complicated data-driven ICA outcomes. It is also noted only five valuable ICs were identified in the present study while the number of ICs was selected at 64 in the ICA analysis. Other ICs were identified as artifactual ones. One of factors might be because beta-band EEG signals were included in the ICA analysis, which have higher probability to be related to motion artifacts. Since phenomena identified in the present study are only reported in lower frequency bands. An alternative way of performing ICA analysis might only use data from lower frequency bands (e.g., theta and alpha bands). Thirdly, the large variation in the performance of subjects, this could be controlled with longer training durations and/or subject dependent workload assessment and assignment.

### Future work

To improve data quality, EEG systems capable of long duration recordings can be used without the need of impedance check (Steriade and Amzica, 2003) and using advanced signal processing techniques for better artifact rejection can aid in reliable identification of neural components. The properties of fast convergence and low computational complexity of online recursive ICA (ORICA) can potentially enable the realization of real-time online ICA process, which further coupled with adaptive ORICA (Hsu et al., 2015) can learn and re-learn task related components. Evaluation of encoded data from these components can assist in the development of real-time adaptive HMI applications to mitigate any lapses in mental capabilities. It is also noted that the factors of mental workload and mental effort cannot be completely alienated and need to be further studied together to understand mental efficiency in operators using a unified metric (Camp et al., 2001). Regarding beta band signal, we will exclude the beta band signal in ICA calculation if we only examine neural markers at theta and alpha bands in the future.

## Conclusion

In conclusion, EEG spectral power correlates to three factors, i.e., mental fatigue, mental effort, and mental workload, are successfully elucidated at the IC domain in a realistic ATC task. Five ICs were successfully disentangled from scalp EEG data with characteristic spectral and spatial patterns. Identified ICs reflected performance monitoring/short-term memory (the frontal IC), motor preparation (the central medial IC), motor execution (the motor IC), sensory information integration/decision making (the parietal IC), and visual perception and process (the occipital IC). The present results indicate that spectral power changes in EEG component signals, especially those having established links to specific neural processes, encode information about various mental factors. The present results further indicate that the ICA method is a promising technique in revealing dynamics of these component signals in continuous EEG data and, therefore, indicating the evolution of mental states during real-world tasks.

## Author contributions

DD: collected the EEG data, analyzed the data, wrote the manuscript. GS: analyzed the data, wrote the manuscript. LD: designed the experiment, wrote the manuscript.

### Conflict of interest statement

The authors declare that the research was conducted in the absence of any commercial or financial relationships that could be construed as a potential conflict of interest.
